# Comparative analysis of early outcomes of the first 150 cases of posterior approach robotic‐assisted radical prostatectomy and identification of the learning curve: A single‐surgeon series

**DOI:** 10.1002/bco2.70058

**Published:** 2025-07-23

**Authors:** Li June Tay, Henry Y. C. Pan, Leigh James Spurling, Philip Dundee

**Affiliations:** ^1^ Department of Urology East Kent Hospital NHS Trust Canterbury UK; ^2^ Department of Urology The Royal Melbourne Hospital Parkville Victoria Australia; ^3^ Department of Surgery The University of Melbourne Parkville Victoria Australia; ^4^ Department of Targeted Intervention University College London London UK; ^5^ Department of Surgery Epworth Hospital Richmond Richmond Victoria Australia

**Keywords:** learning curve, outcomes, prostate cancer, prostatectomy, Retzius sparing, robotics

## Abstract

**Objectives:**

To report intraoperative and early postoperative outcomes of posterior approach Robotic Assisted Radical Prostatectomy (RARP) patients and analyse a single‐surgeon learning curve.

**Patients & Methods:**

The initial 150 patients undergoing posterior approach RARP by a single surgeon were analysed in three equal cohorts. Initial postoperative follow‐up was for a minimum of 3 months.

**Results:**

A total of 150 patients were included. These cases were performed between April 2017 to June 2024. There was no significant difference in pre‐operative patient age, prostate specific antigen (PSA), body mass index (BMI), prostate volume, number of biopsy positive cores, International Society of Urological Pathologists (ISUP) grade group and clinical T‐stage.

Intraoperative differences between cohorts were decreasing total operative time (153 min vs 121 min vs 106 min, p < 0.001) and estimated blood loss (296 ml vs 205 ml vs 199 ml, p < 0.001), but no difference in nerve sparing status (p = 0.243).

Postoperatively, no difference was found in median length of stay, ISUP grade group, tumour volume, 30‐day readmissions or complications. There were significant differences in overall pathological T stage (p = 0.004) between the cohorts, but not positive margin status, even with T2/T3 disease. There was a significant difference in early continence recovery within the first week (p = 0.022) and at 1 month (0.041) but no difference between overall continence recovery and erectile function recovery.

**Conclusions:**

Estimated blood loss and total operative time decreased across the cohorts, despite worsening disease burden. Oncological and functional outcomes are excellent throughout when compared with published literature. The learning curve may be facilitated initially by careful patient selection. Posterior approach RARP could be safely adopted by urologists adept in standard RARP, and structured training may improve the uptake of this technique.

## 1 INTRODUCTION

Prostate cancer is the second most commonly diagnosed cancer in men globally. The World Health Organisation estimates 1.4 million new cases in 2020.^1^ In Australia, it is the most commonly diagnosed cancer among men. The Australian Institute of Health and Welfare projected that in 2024, over 26 000 new cases will be diagnosed with an age standardised rate of 97.1 cases per 100 000 persons.^2^ Robotic assisted radical prostatectomy (RARP), laparoscopic assisted radical prostatectomy and open retropubic radical prostatectomy are currently the options for surgical treatment in localised disease. RARP is now the standard approach since its introduction in the early 2000s, and has been widely adopted, particularly in Europe and North America, with a consistently increasing uptake in Australia.^3^ RARP is usually performed using the standard anterior approach, and there are many formal training programs and fellowships available worldwide to learn this technique.^4^ However, Retzius sparing prostatectomy (RS‐RARP), also known as the posterior approach, is relatively new and was first described by Galfano and colleagues in 2010.^5^ This technique was developed with the aim to achieve better postoperative urinary continence and sexual function by removing the prostate via the rectovesical pouch and preserving the integrity of anteriorly located structures. Several initial studies have highlighted concerns about oncological safety, specifically the higher risk of positive surgical margins (PSM).^6^ This study aims to identify the outcomes of RS‐RARP as the surgeon progresses along the learning curve of this novel technique.

## 2 MATERIALS AND METHODS

### 2.1 Study design

This study was a non‐controlled prospective case series of our first 150 posterior approach RS‐RARP performed by a single surgeon. The study timeframe was between May 2017 and June 2024, cases were highly selected during the initial learning curve, with gradually less selection over time, such that all cases were eventually performed via the posterior approach. Cases were divided chronologically and by volume into three groups, each with 50 cases as follows:

Cohort 1: 50 cases between 19 April 2017 to 5th August 2021; Cases 1–50
Cohort 2: 50 cases between 6th Aug 2021 to 29th June 2023: Cases 51–100
Cohort 3: 50 cases between 30th June 2023 to 6th June 2024: Cases 101–150

### 2.2 Outcome variables evaluated

Pre‐operative patient characteristics include age, body mass index (BMI), prostate‐specific antigen (PSA), erectile function status, prostate volume, International Society of Urological Pathology (ISUP) grade group at time of prostate biopsy, number of positive cores on prostate biopsy, clinical T stage (based on digital rectal examination). Intraoperative characteristics include estimated blood loss (EBL), total operative time, nerve sparing status and pelvic lymph node dissection (PLND) were included.

Postoperative outcomes include general, oncological and functional outcomes. Oncological outcomes include pathological T stage, pathological ISUP grade group, tumour volume, number of lymph nodes sampled, PSM and biochemical recurrence (defined as postoperative PSA > 0.2 ng/ml). General outcomes included were inpatient length of stay, 30‐day unplanned readmissions and complications according to the Clavien‐Dindo classification. Functional outcomes were postoperative urinary continence and erectile function.

Erectile function or potency was defined as the ability to achieve erections sufficient for penetrative intercourse. This data was patient self‐reported and collected at each follow‐up interval. Urinary continence was patient‐reported pad usage numbers with full continence defined as strictly zero pad use and social continence as 0–1 pad per day.

Positive margin status was defined as the presence of tumour at the inked margin on histological analysis.

Margin status and urinary continence are also compared to a standard anterior approach robotic‐assisted prostatectomy performed by the same surgeon during the same period to ensure safety and efficacy.

### 2.3 Surgical technique and case selection

The operating surgeon had completed fellowship training in anterior LRP and RARP and following completion of training, had performed approximately 150 standard anterior approach RARP and laparoscopic (LRP) cases prior to transitioning to RS‐RARP. Almost all cases of posterior approach RS‐RARP were performed in the private sector, with 3 RS‐LRP cases and 3 RS‐RARP public sector cases.

The Intuitive® Da Vinci Xi® (Intuitive Surgical Inc, California, USA) 4 arm robot was used for most robotic cases, with a small number performed on the Intuitive® Da Vinci Si® (Intuitive Surgical Inc, California, USA) 4 arm robot early in the series. During the study time frame, 325 cases of standard anterior RARP and LRP were also completed across both the private and public sectors. LRP was performed at centres without access to a robotic surgical platform with the last case performed in 2020.

Cases excluded from the posterior approach RS‐RARP and performed with a standard anterior approach were those with gland size >80 ml on Magnetic Resonance Imaging (MRI), BMI > 35 kg/m^2^, large median lobe on MRI, anterior index lesion on MRI, or prior transurethral resection of prostate. Selection criteria were gradually loosened with experience such that during the third cohort of 50 cases, all selection criteria were dropped and all cases were performed via the posterior approach in the private sector.

The surgical technique used is similar to that as described by Galfano and colleagues.^7^ A pelvic drain was not routinely inserted. No cases of pelvic collection requiring intervention were recorded. Standard perioperative management included an overnight stay in hospital, early mobilisation, a single dose of low molecular weight heparin postoperatively and the use of simple analgesia as required on discharge.

A suprapubic catheter (SPC) was inserted at the time of surgery. This was left on free drainage. The SPC was clamped during the trial of void at one week. Fluoroscopic cystogram was not performed routinely. If trial of void was successful, the SPC would be removed. Subsequent follow‐up was at 1 month and 3 months, with subsequent follow‐up depending on histopathology results. Low and intermediate risk patients with an undetectable PSA at 3 months were next seen at 12 months and annually thereafter. Higher risk patients and those with a detectable PSA at 3 months were followed more frequently. The minimum follow‐up data for all patients was 3 months in this series.

### 2.4 Data acquisition

All patient information and clinical data were entered into their medical records and prospectively compiled onto a database with institutional ethics approval, which has been maintained by the primary surgeon. De‐identified data required for the case series and the corresponding time period for analysis was extracted onto a study database and analysed using Microsoft Excel.

### 2.5 Statistical analysis

Statistical analysis was performed using R version 4.2.2. Statistical significance was considered where p < 0.05 value. Group‐wise comparison was made using Chi‐squared tests for categorical data; analysis of variance for continuous values and log‐rank test for time to event data. Mean and standard deviation was calculated for continuous variables. A survival analysis of 12‐week continence outcomes, comparing the three cohorts of patients (C1, C2 &C3) was conducted. A Cox proportional hazards model was used for return of continence by 12 weeks.

## 3 RESULTS

The mean patient age was 63.7 years. Over the three cohorts, there was a statistically significant reduction in total operating time (153mins vs 121mins vs 106mins; p < 0.001) and estimated blood loss (296 ml vs 205 ml vs 199 ml; p < 0.001). Only one patient required blood transfusion in the first cohort for postoperative bleeding otherwise managed conservatively. There was no difference between groups in pre‐operative patient characteristics (seen in table 1) including age, BMI, PSA, prostate volume, biopsy according to ISUP Grading System, clinical T stage, number of positive cores on prostate biopsy and pre‐operative erectile function. There was no statistically significant difference between groups for nerve sparing status, pelvic lymph node dissection status, number of lymph nodes sampled, tumour volume, margin status, median length of stay, 30‐day unplanned readmissions or major postoperative complications (Grade ≥3 Clavien‐Dindo).

**TABLE 1:** Pre‐operative patient characteristics and intraoperative variables.

Operative group	Cases 1–50		Cases 51–100		Cases 101–150		*p value*
	*Mean/N*	*Sd/%*	*Mean/N*	*Sd/%*	*Mean/N*	*Sd/%*	
Pre and intra operative characteristics							
Age (years)	63.28	7.49	64	7.59	63.9	9.14	0.891
Body mass index (kg/m2)	26.53	3.37	26.53	3.1	27.78	3.84	0.203
Prostate volume (ml)	34.96	15.26	32.54	11.6	37.64	15.8	0.21
PSA (ng/ml)	6.67	2.97	5.8	4.32	7.29	4.03	0.153
Positive cores on biopsy (*n*)	6.46	3.16	7.64	3.37	6.78	3.25	0.177
Biopsy ISUP Grade Group							
*1*	0	0	3	6	1	2	0.392
*2*	39	78	35	70	28	56	0.424
*3*	7	14	10	20	13	26	0.928
*4*	3	6	2	4	5	10	0.497
*5*	1	2	0	0	3	6	0.435
Clinical T stage							0.697
*1c*	27	54	34	68	32	64	0.702
*2a*	11	22	5	10	8	16	0.426
*2b*	5	10	4	8	0	0	1
*2c*	0	0	1	2	0	0	1
*3a*	7	14	5	10	9	18	0.693
*3b*	0	0	0	0	1	2	0.317
*Not recorded*	0	0	1	2	0	0	
Existing erectile dysfunction	6	12	6	12	2	4	0.484
Pelvic lymph node dissection	0	0	1	2	0	0	n/a
Nerve Spare							0.352
*None*	1	2	0	0	1	2	
*unilateral*	2	4	2	4	2	4	
*bilateral* (*full*/*incremental*)	17	34	8	16	13	26	
*bilateral* (*full*)	30	60	40	80	34	68	
Operative time (minutes)	152.8	26.59	121	18.07	106	21.67	<0.001*
Estimated blood loss (ml)	296	174.33	205	113.35	199	131.9	<0.001*
transfusion	1	2	0	0	0	0	n/a

* = significant at p < 0.05.

### 3.1 Clavien‐Dindo complications

There was no significant difference in Clavien‐Dindo Grade ≥3 between the three cohorts. In the first cohort, there were 4 patients with Grade 3a or 3b complications, all of whom required a flexible or rigid cystoscopy due to urinary retention or ongoing urinary symptoms. There were none noted in the second cohort. In the third cohort, one patient required a return to theatre secondary to small bowel obstruction due to an incarcerated hernia at the specimen extraction site in the left iliac fossa. The bowel was viable at the time of exploration, and the hernia was reduced successfully. However, the patient subsequently developed an incarcerated left inguinal hernia, which was repaired by a general surgeon.

### 3.2 Urinary continence

**FIGURE 1:**
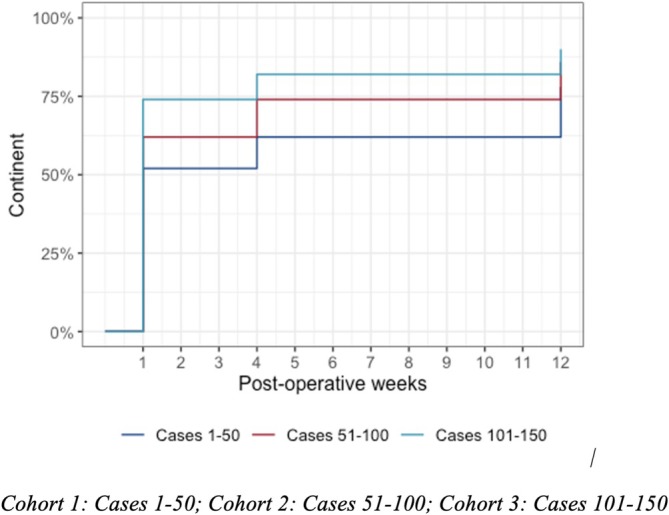
Survival curve of patients achieving full continence in the first 3 months.

**FIGURE 2:**
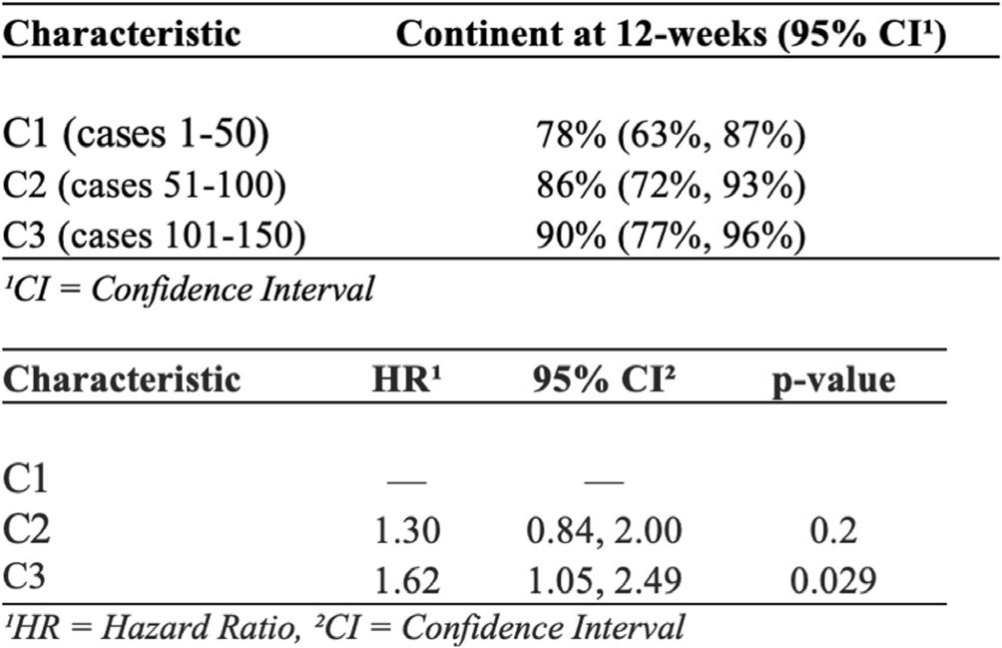
Summary of full urinary continence at 12 weeks by cohort and corresponding 95% CI; and Cox proportional hazards model of early urinary continence by cohort.

**FIGURE 3:**
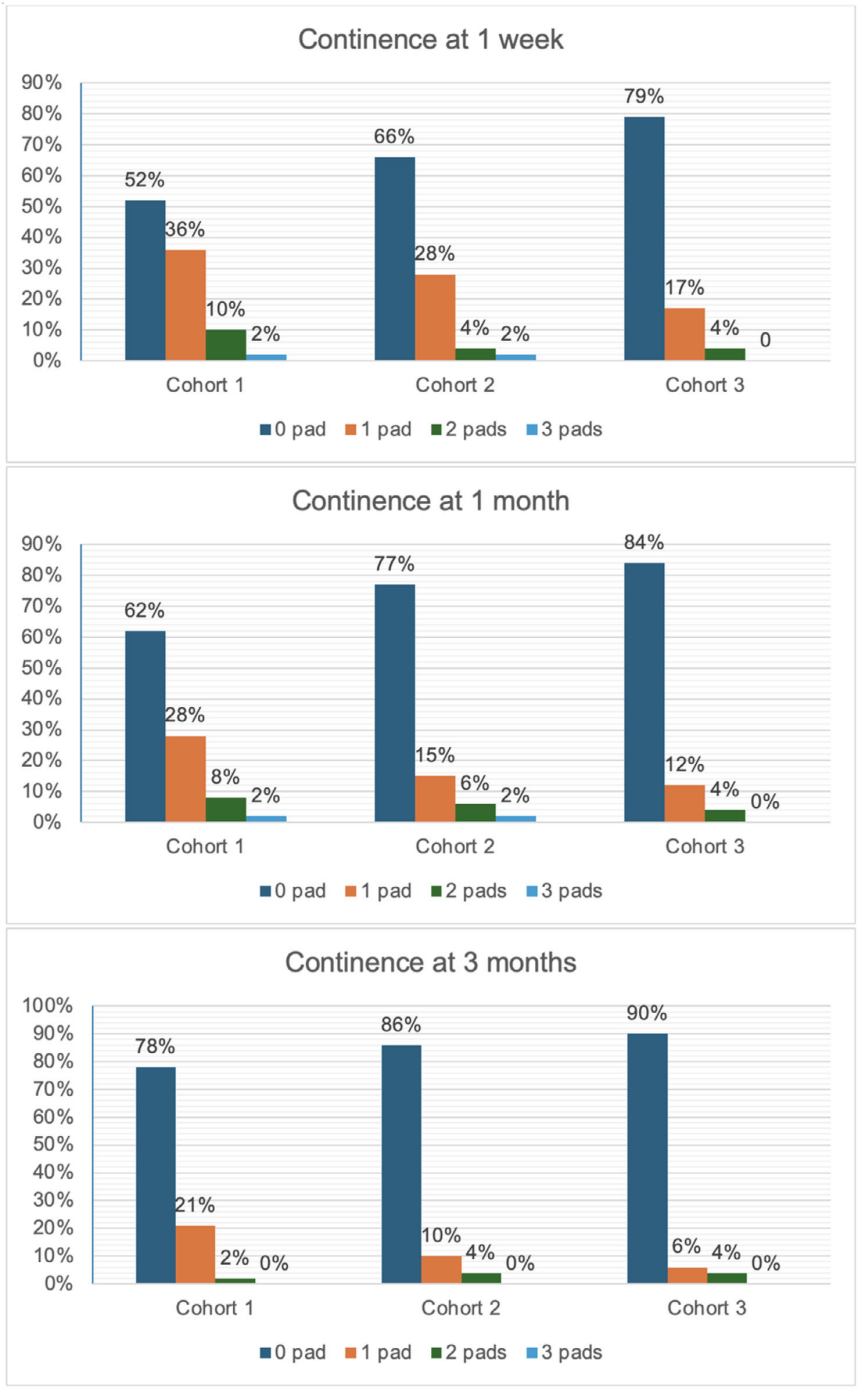
Urinary continence at 1 week, 1 month and 3 months, according to number of pad usage.

There was no statistically significant difference in urinary continence across the 3 groups, and this was achieved by > 90% of patients overall. Early (12 weeks) continence analysis revealed that full urinary continence in C1, C2 and C3 was achieved in 78% (95% CI 63–87%), 86% (CI 72–93%) and 90% (CI 77–96%) of patients respectively (Figure 1, figure 2). The proportion of pad usage were calculated at three time points: 1 week postoperatively (at trial of void), 1 month postoperative and 12 weeks postoperative. Results of the three cohorts are summarised in Figure 3. Statistical analysis revealed a significant difference between cohorts in urinary continence achieved at 1 week (p = 0.022) and at 1 month (p = 0.041) but there was no difference at 12 weeks (p = 0.202). Over the first 12 weeks, patients within C3 were more likely to regain continence (HR 1.62, 95% CI 1.05, 2.49; p = 0.029) compared to the baseline hazards group C1 (Figure 2).

Social continence at 12 weeks (defined as 0–1 pad per day) was achieved in C1, C2 and C3 at 98%; 96% and 96% respectively (p = 0.828).

Overall, there was an obvious improvement in the early continence outcomes at 3 months in the posterior approach RARP group (77% vs 56%) when compared to anterior RARP group during the early learning curve, rising to 90% in the third cohort (90% vs 49%). Social continence for posterior approach RS‐RARP group was more than 95% across all three cohorts.

### 3.3 Erectile function

The overall potency rate across the cohorts was 61.33% and when compared were not statistically significant (52% vs 74% vs 58%; p = 0.17). However, average times to achieving erectile function were 7.3 vs 5.3 vs 3.2 months; p = 0.01. Bilateral nerve spare was performed in 94.7% of patients across the three cohorts, of which 69.3% had bilateral full intrafascial nerve spare (60%; 80%; 68%).

### 3.4 Oncological outcomes

Overall, there was a significant difference in pathological T stage between the three cohorts (p = 0.004). The third cohort had the highest proportion of patients with high risk disease according to ISUP grade. ISUP Grade Group 5 was present in one patient (2%) in C1, two patients (4%) in C2 and eight patients (16%) in C3. On pathological T stage analysis, T3a disease was present in 44% of C1 (n = 22); 20% of C2 (n = 10) and 44% of C3 (n = 22), p = 0.468; and T3b in 8% of C1 (n = 4); 4% of C2 (n = 2) and 6% of C3 (n = 3), p = 0.535.

The overall PSM rate was 10.6%. Analysis of margin status across groups showed no significant difference between groups despite higher risk patients in the third cohort: C1 n = 4, 8%; C2 n = 7, 14%; C3 n = 5, 10%, p = 0.485. When margin status was analysed based only on pathological stage pT2 versus ≥pT3, most PSMs occurred in patients with ≥pT3 disease. Those with pT2 disease had no positive margins in C1 and C3, but 2 patients in C2 (5.3%), compared to patients with ≥pT3 disease (C1: n = 4, 15.4%; C2: n = 5; 41.7% and C3: n = 5; 20% p = 0.795). In total 63 patients had ≥pT3 disease of which 14 had PSM, giving us an average positive margin rate of 22.22% in this group. In the 87 patients with pT2 disease, only two patients (2.3%) in cohort 2 had a PSM. All patients with pT2 disease in Cohort 1 and 3 had negative margins.

Biochemical recurrence occurred in a total of 17 patients. There were 12 patients in C1 (24%), 3 patients in cohort 2 (6%) and 3 patients in cohort 3 (4%). Statistical analysis for significance was not performed because the follow‐up duration varied greatly between the three cohorts.

The above results are summarised and outlined in Table 2.

**TABLE 2:** Postoperative characteristics and outcomes divided by cohorts.

Operative group	Cases 1–50		Cases 51–100		Cases 101–150		P value
	Mean/N	Sd/%	Mean/N	Sd/%	Mean/N	Sd/%	
Postoperative characteristics							
Clavien Dindo ≥3	4	8	0	0	1	2	0.171
Median length of stay (days)	1		1		1		0.069
Pathological ISUP Grade Group							0.159
1	0	0	0	0	0	0	n/a
2	33	66	34	68	29	58	0.537
3	15	30	14	28	11	22	0.467
4	1	2	0	0	2	4	0.355
5	1	2	2	4	6	12	0.812
Tumour volume (ml)	4.46	3.36	3.92	2.75	4.79	3.70	0.472
Lymph nodes sampled (n)	0		7		0		n/a
pathological stage							0.004*
pT2	24	48	38	76	25	50	0.073
pT3a	22	44	10	20	22	44	0.468
pT3b	4	8	2	4	3	6	0.535
Margin status							0.4509
T2	0	0	2	5.3	0	0	0.264
T3	4	15.4	5	41.7	5	20	0.795
Recurrence	12	24	3	6	2	4	n/a
Mean time to recurrence (months)	32.41		10.17		3.04		n/a
Functional outcomes							
Continence (overall)	47	94	45	90	47	96	0.485
Continence (12 weeks)	37	77	42	86	45	90	0.202
Surgery required	1		0		0		n/a
Erections	26	60.5	37	80.4	29	64.4	0.168
Mean time to achieve potency (months)	7.3	6.99	5.3	6.41	3.2	2.49	0.01

* = significant at p < 0.05.

## 4 DISCUSSION

The aim of this study was to describe the outcomes of our first 150 cases of robotically assisted Retzius sparing prostatectomy and to compare outcomes between the first, second and third cohorts of 50 patients in our series in order to evaluate the learning curve. Additionally, we compared our outcomes to the published literature, and to our own cohort of patient who underwent standard anterior approach RARP in the same time period with the same surgeon to ensure that the adoption of this technique was beneficial and safe for patients compared to the standard treatment.

Although these patient and disease factors did not reach statistical significance between the 3 cohorts, the general trend skewed towards larger gland size, higher BMI, larger tumour volume and higher T stage in the third cohort of patients compared to the first two cohorts, reflecting the progressive relaxation of patient selection criteria along the learning curve. There was a statistically significant difference in operative time and estimated blood loss between the three cohorts. These parameters were the highest in the first cohort and declined across the three cohorts. This was despite the increasing case complexity in the third cohort to include larger gland size, prostates with significant median lobe, more advanced disease including anterior index lesions on MRI and a patient with prior pelvic radiation for a rectal malignancy.

Despite the improved early continence, overall adoption of the Retzius sparing technique has been slow due to the perceived risk of positive margins, which in early series was higher compared to the anterior approach.^8^, ^9^ However, from our study, it is reassuring that PSM rates were not significantly different between the three groups. Across three cohorts, the PSM rates for pT2 disease was 2.3% (range 0–5.3%), which is lower than reported in the literature,^7^, ^10^ while the PSM for locally advanced pT3 disease was 22.2%, (range15–41%) which is consistent with the published literature.^10^ Given the concerns of PSM especially in the anterior prostate,^9^ we excluded cases with an anterior index lesion on MRI during the early learning curve in cohort 1. When compared with our standard RARP cohort, which was performed concurrently during the same time period and in which the overall PSM was 18%, the overall PSM was 10.7% in this cohort. It should be noted that the standard RARP cohort included patients with worse disease and more unfavourable patient factors initially. When sub‐analysed into patients with pT2 versus ≥ pT3 disease, the PSM rate for pT3 disease in the anterior RARP group was 28.57% versus 22.22% in this RS‐RARP cohort. It was reassuring that the PSM rate was no worse in the RS‐RARP group and supports the adoption of this technique without compromise in oncological safety.

Recent literature^11^ including randomised controlled trials^12^ has concluded that there is no significant difference in the biochemical recurrence‐free survival (bRFS) between patients who had standard RARP and RS‐RARP in the early and intermediate term. However, one of the major limitations of this study is the discrepancy of follow‐up durations between the three cohort. Especially given that the minimum follow‐up duration was three months, which is predominantly applicable to patients in the third cohort, we lack data regarding bRFS. We did not compare this as the follow‐up period was not long enough to draw any meaningful conclusions. In our first 50 cases, the average time to recurrence was 32.4 months. Further studies of our cohort would be required to evaluate intermediate and longer term bRFS.

The learning curve for RS‐RARP is thought to be longer and more difficult than standard RARP due to the restricted working space between the bladder and rectum and the lack of anatomical landmarks. However, there are few studies that have formally assessed this. In addition, training for this technique tends to be informal, self‐taught and ad‐hoc, which is our primary surgeon's experience, and this is a largely similar experience with other contemporary series.^13^

This is a single‐surgeon series and the findings may not be generalised. The surgeon had undertaken 150 cases of standard RARP or LRP prior to adopting this technique and continued to perform standard RARP during the adoption of the Retzius sparing approach. However, there is evidence that even in less experienced hands that early oncological outcomes are still maintained.^14^

One of the main advantages of the Retzius sparing technique is the early continence recovery.^9^, ^15^, ^16^ In our series, we have also shown superior results compared to standard RARP cases performed during the same time period. Continence recovery was also shown to improve throughout the learning curve, where there was a significant difference in continence rates at 1 week and 1 month postoperative, but at three months this had diminished. Overall the last cohort was more likely to regain continence sooner compared to the first cohort. Our series showed that 90% of the last cohort of RS‐RARP were continent at 3 months compared to just over half of those in our standard RARP group. In addition, even at one year, our standard RARP group's continence recovery was at 80%. This is based on the definition of continence as strictly 0 pads per day.^17^ With a more liberal definition of “social continence” including patients wearing a safety pad or single pad for minor leakage, the rates were up to 95% for the third group of RS‐RARP patients at trial of void (1 week postoperative) and 96% at 3 months, which is superior compared to the standard RARP where social continence was 90% at 3 months.

There has not been any established benefit of RS‐RARP over standard RARP with regards to sexual function. A recent meta‐analysis showed no difference in return of erectile function in men who had undergone RS‐RARP compared to standard RARP.^8^ Conversely, other parameters such as patient reported penile shortening and Peyronie's Disease was lower in RS‐RARP compared to standard RARP.^18^ In our RS‐RARP series, potency is achieved in 61.3% of patients, with no significant difference between the three cohorts. This is superior compared to patients in our standard RARP cohort performed during the same time frame, where only 32% were potent at the latest follow‐up. Additionally, we noted a statistically significant improvement in the average time to achieve potency across the cohorts, which reduced from an average of 7.3 months to 3.2 months. However, we acknowledge that our erectile function were collected through patient self‐reported outcomes and not evaluated using standardised validated questionnaires such as the International Index of Erectile Function (IIEF). This is due to difficulty collecting questionnaire data particularly where follow‐ups were mostly done as telehealth consultations, especially since the COVID‐19 pandemic, in addition to limited research resources in a private healthcare setting.

Finally, this study is non‐controlled in nature. Patient selection criteria was more stringent for the first 50 cases of the learning curve, such as selecting those with low volume disease and smaller gland size, whereas as the experience of the surgeon increased, patients with more advanced disease and larger glands were included. Hence there may be a bias in case selection although our analysis did not show any statistically significant differences in these factors between the cohorts.

## 5 CONCLUSION

Despite informal training for the RS‐RARP technique, our series has shown promising early functional and oncological outcomes, despite being early in the learning curve. We found that the overall PSM rates did not change across the learning curve and remained at least equivalent to contemporary series of both RS‐RARP and standard RARP. In addition, the early continence outcomes improve across the learning curve, and are superior compared to standard RARP, with almost all patients achieving full continence within 3 months. Further studies are required to assessed intermediate and long‐term oncological outcomes as well as potency outcomes. RS‐RARP technique can be safely adopted by surgeons who are fluent and competent in standard RARP techniques. As more surgeons adopt this technique, a formalised training program would be beneficial for surgeons seeking to learn RS‐RARP, and fellowship programs can be adapted to include both techniques of RARP.

## AUTHOR CONTRIBUTIONS

*Conceptualisation*: Philip Dundee. *Original draft*: Li June Tay, Henry Y. C. Pan and Philip Dundee. *Data curation and analysis*: Li June Tay, Henry Y. C. Pan and Leigh James Spurling. *Review and Editing*: All authors.

## CONFLICT OF INTEREST STATEMENT

The authors have no relevant financial or non‐financial interests to disclose.

## References

[1] Bray F , Laversanne M , Sung H , Ferlay J , Siegel RL , Soerjomataram I , et al. Global cancer statistics 2022: GLOBOCAN estimates of incidence and mortality worldwide for 36 cancers in 185 countries. CA Cancer J Clin. 2024;74(3):229–263. 38572751 10.3322/caac.21834

[2] Australian Institute of Health and Welfare . Cancer data in Australia [internet] Canberra: AIHW; 2024 Available from: https://www.aihw.gov.au/reports/cancer/cancer-data-in-australia

[3] Maynou L , Mehtsun WT , Serra‐Sastre V , Papanicolas I . Patterns of adoption of robotic radical prostatectomy in the United States and England. Health Serv Res. 2021;56(Suppl 3):1441–1461. 34350592 10.1111/1475-6773.13706 PMC8579206

[4] Martini A , Falagario UG , Villers A , Dell'Oglio P , Mazzone E , Autorino R , et al. Contemporary techniques of prostate dissection for robot‐assisted prostatectomy. Eur Urol. 2020;78(4):583–591. 32747200 10.1016/j.eururo.2020.07.017

[5] Galfano A , Ascione A , Grimaldi S , Petralia G , Strada E , Bocciardi AM . A new anatomic approach for robot‐assisted laparoscopic prostatectomy: a feasibility study for completely Intrafascial surgery. Eur Urol. 2010;58(3):457–461. 20566236 10.1016/j.eururo.2010.06.008

[6] Ferretti S , Dell'Oglio P , Ciavarella D , Galfano A , Schips L , Marchioni M . Retzius‐sparing robotic‐assisted prostatectomy: technical challenges for surgeons and key prospective refinements. Res Rep Urol. 2023;15:541–552. 38106985 10.2147/RRU.S372803 PMC10725648

[7] Galfano A , Di Trapani D , Sozzi F , Strada E , Petralia G , Bramerio M , et al. Beyond the learning curve of the Retzius‐sparing approach for robot‐assisted laparoscopic radical prostatectomy: oncologic and functional results of the first 200 patients with ≥1 year of follow‐up. Eur Urol. 2013;64(6):974–980. 23856036 10.1016/j.eururo.2013.06.046

[8] Barakat B , Othman H , Gauger U , Wolff I , Hadaschik B , Rehme C . Retzius sparing radical prostatectomy versus robot‐assisted radical prostatectomy: which technique is more beneficial for prostate cancer patients (MASTER study)? A systematic review and meta‐analysis. Eur Urol Focus. 2021. 10.1016/j.euf.2021.08.003 34429272

[9] Checcucci E , Veccia A , Fiori C , Amparore D , Manfredi M , Di Dio M , et al. Retzius‐sparing robot‐assisted radical prostatectomy vs the standard approach: a systematic review and analysis of comparative outcomes. BJU Int. 2020;125(1):8–16. 31373142 10.1111/bju.14887

[10] Patel VR , Coelho RF , Rocco B , Orvieto M , Sivaraman A , Palmer KJ , et al. Positive surgical margins after robotic assisted radical prostatectomy: a multi‐institutional study. J Urol. 2011;186(2):511–517. 21680001 10.1016/j.juro.2011.03.112

[11] Egan J , Marhamati S , Carvalho FLF , Davis M , O'Neill J , Lee H , et al. Retzius‐sparing robot‐assisted radical prostatectomy leads to durable improvement in urinary function and quality of life versus standard robot‐assisted radical prostatectomy without compromise on oncologic efficacy: single‐surgeon series and step‐by‐step guide. Eur Urol. 2021;79(6):839–857. 32536488 10.1016/j.eururo.2020.05.010

[12] Barayan GA , Majdalany SE , Butaney M , Dalela D , Peabody J , Abdolla F , et al. Intermediate‐term oncologic outcome assessment for robot‐assisted radical prostatectomy: comparing Retzius‐sparing with standard approach in a randomized control cohort. J Endourol. 2024;38(6):559–563. 38429913 10.1089/end.2023.0514

[13] Hussein H , Maitra N , Tay LJ , Saxionis I , Makin R , Sivathasan S , et al. Analysis of the learning curve for Retzius‐sparing robot‐assisted radical prostatectomy for a single surgeon. J Clin Urol. 2023;18(3):231–239. 10.1177/20514158231203766

[14] Olivero A , Galfano A , Piccinelli M , Secco S , Di Trapani D , Petralia G , et al. Retzius‐sparing robotic radical prostatectomy for surgeons in the learning curve: a propensity score‐matching analysis. Eur Urol Focus. 2021;7(4):772–778. 32192919 10.1016/j.euf.2020.03.002

[15] Dalela D , Jeong W , Prasad MA , Sood A , Abdollah F , Diaz M , et al. A pragmatic randomized controlled trial examining the impact of the Retzius‐sparing approach on early urinary continence recovery after robot‐assisted radical prostatectomy. Eur Urol. 2017;72(5):677–685. 28483330 10.1016/j.eururo.2017.04.029

[16] Umari P , Eden C , Cahill D , Rizzo M , Eden D , Sooriakumaran P . Retzius‐sparing versus standard robot‐assisted radical prostatectomy: a comparative prospective study of nearly 500 patients. J Urol. 2021;205(3):780–790. 33086025 10.1097/JU.0000000000001435

[17] Liss MA , Osann K , Canvasser N , Chu W , Chang A , Gan J , et al. Continence definition after radical prostatectomy using urinary quality of life: evaluation of patient reported validated questionnaires. J Urol. 2010;183(4):1464–1468. 20171689 10.1016/j.juro.2009.12.009

[18] Kowalczyk KJ , Davis M , O'Neill J , Lee H , Orzel J , Rubin RS , et al. Impact of Retzius‐sparing versus standard robotic‐assisted radical prostatectomy on penile shortening, Peyronie's disease, and inguinal hernia sequelae. Eur Urol Open Sci. 2020;22:17–22. 34337474 10.1016/j.euros.2020.09.004 PMC8317841

