# Correction to ‘Comparative analysis of early outcomes of the first 150 cases of posterior approach robotic‐assisted radical prostatectomy and identification of the learning curve: A single‐surgeon series’

**DOI:** 10.1002/bco2.70199

**Published:** 2026-04-10

Tay LJ , Pan HYC , Spurling LJ , Dundee P . Comparative analysis of early outcomes of the first 150 cases of posterior approach robotic‐assisted radical prostatectomy and identification of the learning curve: A single‐surgeon series. BJUI Compass. 2025;6(7):e70058. 10.1002/bco2.70058 40708758 PMC12286756

Authors Tay and Pan contributed equally and are co‐first authors. This should have been noted in the published version.

We apologise for this error.

